# Exploring perspectives on restraint during medical procedures in paediatric care: a qualitative interview study with nurses and physicians

**DOI:** 10.1080/17482631.2017.1363623

**Published:** 2017-09-11

**Authors:** Edel Jannecke Svendsen, Reidar Pedersen, Anne Moen, Ida Torunn Bjørk

**Affiliations:** ^a^ Department of Nursing, Institute of Health and Society, Faculty of Medicine, University of Oslo, Oslo, Norway; ^b^ Centre for Medical Ethics, Institute of Health and Society, Faculty of Medicine, University of Oslo, Oslo, Norway

**Keywords:** Restraint, child, children’s nurses, symbolic interactionism, qualitative study, hospitalised child

## Abstract

The aim of this study was to explore nurses’ and physicians’ perspectives on and reasoning about the use of restraint during medical procedures on newly admitted preschoolers in somatic hospital care.

We analysed qualitative data from individual interviews with a video recall session at the end with seven physicians and eight nurses. They had earlier participated in video recorded peripheral vein cannulations on preschool children. The data were collected between May 2012 and May 2013 at a paediatric hospital unit in Norway.

The analysis resulted in three main themes: (1) disparate views on the concept of restraint and restraint use (2), ways to limit the use of physical restraint and its negative consequences, and (3) experience with the role of parents and their influence on restraint. Perspectives from both healthcare professions were represented in all the main themes and had many similarities.

The results of this study may facilitate more informed and reflective discussions of restraint and contribute to higher awareness of restraint in clinical practice. Lack of guidance and scientific attention to restraint combined with conflicting interests and values among healthcare providers may result in insecurity, individual dogmatism, and a lack of shared discussions, language, and terminology.

## Introduction

Children often undergo potentially painful and frightening medical procedures in hospitals and can experience distress, pain, and anxiety and may express strong and persistent resistance during procedures (Söderbäck, 2013). Restraint seems to be more frequently used with pre-schoolers during different medical and clinical procedures than with older children (Crellin et al., 2011) and is used to enable safe performance of the medical procedure when the child resists it. The use of restraint to accomplish the medical procedure may worsen the child’s experiences (Brenner, 2007; Snyder, 2004) and is potentially harmful and traumatizing. Healthcare providers often collaborate to perform medical procedures on children (Brenner, Treacy, Drennan, & Fealy, 2014; Crellin et al., 2011; Demir, 2007; Kangasniemi, Papinaho, & Korhonen, 2014). Potential challenges are related to healthcare providers’ double roles as appliers of restraint and providers of safe treatment, comfort, and care (Babl et al., 2012). Restraint can be challenging but has been sparingly investigated in paediatric practice (Bray, Carter, & Snodin, 2016). Furthermore, few studies have explored both physicians’ and nurses’ perspectives in these situations. How healthcare providers comprehend different aspects of restraint is important for understanding how restraint is used and for identifying possible solutions when children resist medical treatment and care. In this study, peripheral vein cannulation (PVC), a common medical procedure, in an acute paediatric unit was used as the example in the exploration of healthcare providers’ perspectives and reasoning on restraint.

## Background

Recent research articles use the terms “restraint,” “holding,” or “restriction” to refer to restraint or coercive actions in the paediatric setting (Crellin et al., 2011; Demir, 2007; Kangasniemi et al., 2014; Page & McDonnell, 2013). The different terms imply that the content and naming of these practices are unclear. There is a lack of clear and agreed terminology and nurses and allied healthcare providers differ in their description of their practices (Kirwan & Coyne, 2016; Page & McDonnell, 2013). It is uncertain if the terms cover the same or different aspects of what is going on (McGrath, Forrester, Fox-Young, & Huff, 2002). In one respect, the terms are related to the amount of physical force needed to enable a procedure, such as the difference between holding and restraint (Brenner et al., 2014; Jeffery, 2010). For example, Crellin et al. (2011) suggested grading restraint as none, gentle, moderate, and forceful, based on how large a part of the child’s body (for example, the torso and the number of limbs) was held and the amount of force used when holding them. “Holding” can be, but is not always, voluntary. Holding represents the action of restraint and can be forceful but also kind and loving. The term holding is less specific than the term restraint. The term “restraint” perhaps more clearly refers to a lack of voluntariness compared to “holding” and “restriction.” Restraint in this paper is understood as “the application of force with the intention of overpowering the child, and is by definition applied without the child´s consent” (Royal College of Nursing, 2003, p. 4).

Parents’ presence during medical procedures is valued and expected by healthcare providers because it may reduce distress and worry and can help comfort the child (Cavender, Goff, Hollon, & Guzzetta, 2004; Gilboy & Hollywood, 2009; Snyder, 2004). Healthcare providers often take parental participation in restraint for granted (Hallström, Runeson, & Elander, 2002) and there are indications that parents provide most of the holding during procedures (Graham & Hardy, 2004; Homer & Bass, 2010; McGrath & Huff, 2003). The way healthcare providers understand the parents’ role and cooperate with them during restraint is important because it may be an opportunity to prevent restraint. However, the parents’ wish to participate may differ (Hallström & Elander, 2004; Hallström et al., 2002; Lam, Chang, & Morrissey, 2006). Some parents have reported holding their children as meaningful (Sparks, Setlik, & Luhman, 2007), while other parents find it emotionally difficult (Alexander, Murphy, & Crowe, 2010; Hallström & Elander, 2004; Idvall, Holm, & Runeson, 2005; McGrath & Huff, 2003). It is unclear what is in the child’s best interest.

Previous studies mainly investigated nurses’ perspectives on restraint. Nurses had mixed perspectives and emotions related to restraint (Brenner et al., 2014; Gilboy & Hollywood, 2009; Snyder, 2004). Some nurses had problems with accepting the use of restraint and felt that restraint could harm the relationship they tried to build with paediatric patients (Bricher, 1999; Svendsen & Bjørk, 2014). Nurses experienced having to balance diplomacy with use of restraint (Karlsson, Rydstrom, Enskar, & Englund, 2014) and respond to non-adherence with persuasion and coercion (Kangasniemi et al., 2014). Some nurses who concluded that restraint was often the only way to manage children and to enable medical procedures (Kangasniemi et al., 2014) viewed restraint in some form as inevitable and acceptable (Brenner et al., 2014; Kangasniemi et al., 2014). Kangasniemi et al. (2014) found that nurses considered restraint important because it eased their work and fulfilled the aim of good nursing care because restraint was held to be best for the patient. In other studies, nurses who saw restraint as unacceptable could find it difficult to choose between causing harm and promoting health (Ives & Melrose, 2010; Lloyd, Urquhart, Heard, & Kroese, 2008; McGrath & Huff, 2003). Delaney (2001) performed an ethical analysis of nurses’ perspectives on the harm of restraint versus the benefit in psychiatric settings and concluded that holding a child was perceived as “reasonable harm” compared with the benefit of the treatment.

Use of restraint is not usually specifically mentioned in legislation, although some countries may require parents’ signatures (Demir, 2007). Coercive medical treatment for minors is generally neither an issue in international clinical guidelines nor in the law regulating the practice in Norway, where this study was performed (Stock, Hill, & Babl, 2012; Troianos et al., 2011). However, the main rule in Norwegian health law, as in international human rights guidelines, is that any use of coercion requires an explicit legal authority, a formal decision, and appeal procedures. The lack of clear guiding principles for when and how to use restraint can create professional and ethical challenges for healthcare providers and may influence their clinical judgments (Ives & Melrose, 2010). Since restraint in this setting is not specifically regulated and generally not accompanied by a formal decision and documentation, the restraint used can be defined as “informal” restraint. To develop more knowledge about informal restraint in paediatric healthcare and facilitate more open discussions, this paper explores healthcare providers’ perspectives on restraint.

This study was inspired by symbolic interactionism (SI); SI provides perspectives on how people seek to understand the meaning of others’ actions in a social interaction (Blumer, 1969). Humans act toward people or things based on how they assign meaning to them. Meanings are assigned as symbols. For example, within a situation, one can see another person as uncooperative or lazy. Such symbols (for example, uncooperative) are assigned to others within a social situation, such as during a medical procedure. According to SI, we act toward people as if those symbols of meaning exist. Individually and collectively, people act based on the meanings things have for the individuals, and these meanings arise and are learned in interactions (Burbank & Martins, 2010). In social situations, the symbols (such as the language used) are developed during previous interactions. According to SI, such meanings are assigned and modified through an interpretive process that is always changing and where the meaning is subject to redefinition (Blumer, 1969). People attach certain common meanings to social positions (i.e., nurse or parent); thus, people expect a specific kind of conduct and behaviour from people in these positions. Accordingly, an individual who occupies the position is often aware of these expectations and the way in which he or she is viewed and may act in the roles he or she is given (i.e., uncooperative) in a situation. Based on this, people form meanings and develop specific ways to respond.

The aim of this study was to explore nurses’ and physicians’ perspectives on and reasoning about the use of restraint during medical procedures on newly admitted preschoolers in somatic hospital care. Knowledge about healthcare providers’ perspectives and reasoning can advance and nuance our understanding of the practices of restraint during medical procedures. The following research questions were developed:

● How do paediatric nurses and physicians participating in restraint practices during medical procedure define restraint?

● How do paediatric nurses and physicians reason about episodes of restraint?

## Methods

The present study is part of a larger research project exploring the use of restraint during medical procedures in preschool children. More specifically, we focused on restraint with children who required a sub-urgent medical procedure with limited time available for planning the medical procedure (this meant that the procedure could be postponed for a limited time, typically some hours, because the child’s condition was not critical). In previously published articles, we have reported on children’s expressions of resistance and on the interaction between healthcare providers and parents (Svendsen, Moen, Pedersen, & Bjørk, 2015, 2016). In this study, we present results from interviews with participating nurses and physicians. We also interviewed the participating parents, and the results from these interviews will be presented in future publications. This study had an explorative qualitative design, which is appropriate when little is known about a phenomenon and one wants to understand people’s views and experiences (Polit & Beck, 2008).

### Participants and setting

The nurses and physicians who participated in this study were sampled during the larger research project. They had recently consented to and participated in a total of 14 video recorded attempts to insert PVC on six inpatient preschool children (aged between 3 and 5 years old). The participating healthcare providers were video recorded during PVC on one child (apart from one physician who was video recorded two times with two different children). More accurate information about how this was done is reported in two earlier studies (Svendsen, Moen, Pedersen & Bjørk, 2015, 2016). Parents and healthcare providers used different levels of force together in the holding of the child during the insertion of the PVCs. The level of restraint ranged from targeted restraint by one nurse holding one child’s hand who showed weak resistance, to forceful restraint of a child who exhibited major resistance while two nurses used a lot of force to hold the torso and all limbs. Eight nurses (aged 26–46 years) and seven physicians (aged 32–44 years) agreed to participate. All except one were female. Their experience providing hospital care to children ranged between 1 and 8 years (apart from one physician who had only 2 weeks of experience). The study’s setting was a medical unit in a large teaching hospital in the southern region of Norway. The medical unit treated children from 0 to 18 years admitted for various medical somatic conditions.

### Data generation

Data were collected between May 2012 and May 2013. The interviews were performed at the hospital in a separate room as soon as possible after we had observed and video-recorded the participants in the procedure. The first author conducted face-to-face semi-structured individual interviews, which are suitable when investigating how people reason about their practice and make meaning of their experiences (Kvale & Brinkmann, 2009). The interviews took place during working hours, were tape-recorded, and lasted between 47 and 108 min.

The interview guide was based on results from earlier research, experiences from the first author’s practice as a paediatric nurse, and incidents observed during the recorded video observations. Themes and questions used in this paper involved the following thematic areas: (1) terms the participants used in their reasoning and considerations about restraint, (2) perspectives on the child/child preparations, (3) the parents’ situation and role during the procedure, and (4) cooperation and discussion with colleagues. Before asking questions from the thematic interview guide, the interviewer asked the participants to talk about their experiences during the recent PVC. Then the interviewer followed up on this first question and initiated conversation about their reflections and understanding of the situation, covering the four themes. Some questions were asked of all healthcare providers, while some came up during one interview and were included as questions in subsequent interviews.

The participants were encouraged to share their thoughts about the recent PVC and previous situations. Knowing that restraint could be a “moral sore spot,” the term “restraint” itself was used with care. However, the term was introduced by both the interviewer and the interviewees and served as part of the exploration. In most cases this enriched the discussions and reflections on the concept. After encountering disapproval about the concept of restraint, the interviewer became even more sensitive to the interviewees’ own definitions of restraint. The interview climate differed and this was included as an aspect during the analysis. For example, when one of the participants answered only with short sentences and rational-based judgments and questioned the value of the research, this was interpreted as tension related to the subject of the research. This tension could also be related to other unidentified reasons.

The participants were offered the opportunity to review the video recording of the PVC they had taken part in. Of the six physicians and six nurses who were asked to view the video recording, five physicians and four nurses accepted. Three declined to watch the recording due to time constraints or an expressed aversion to watching themselves on video. Unfortunately, there were technical problems with the remaining three video recordings, so these participants did not watch it. Such video-recall sessions elicit participants’ subjective understandings of their actual interaction, which is valuable when using theories such as symbolic interactionism (Welsh & Dickson, 2005). The video was shown toward the end of the interview to first capture the participants’ inner experience of the situation before they had the chance to watch themselves from the “outside.” The intention was to help the participants reflect on relevant perspectives when observing their interactions afterward.

### Ethical considerations

The study was approved by the Regional Ethics Committee, REK Southeast C (reference number 2011/2193). Information about the purpose of the larger study was included in the written consent, and participants knew the study explored the use of restraint.

To ensure voluntary participation and avoid researcher pressure to participate, a nurse working in the unit made initial contact with potential participants, informed them orally, and distributed the written consent. The first author asked the participants to confirm their willingness to participate before the interviews with the video-recall procedure started. The participants were guaranteed that their contributions were anonymized. All participants signed a written consent form.

### Analysis

The tape-recorded interviews were transcribed verbatim using Nvivo10®. The first author read all the interviews several times, while the co-authors read parts of the interviews. The transcripts were organized into text parts that represented one assumption, meaning, or reasoning. These text parts were clustered into more than 30 fine-grained subcategories. Examples of subcategories include “emotional parents do not cooperate with us,” “confident parents make confident children,” and “the importance of parents being on our side to avoid restraint.” Initially, we used italics for all the physicians’ transcripts and non-italics for the nurses’ transcripts to keep an overview of how the professional groups were represented in each category. This first context of interpretation reflected the participants’ self-understanding and formed the basis for identification and exploration of commonalities and differences among the different categories.

The second context of analysis followed the suggestions by Kvale and Brinkmann (2009) and was a critical common-sense understanding of the data and included a wider frame of context than that of the subjects themselves. We compared the different categories, going back and forth between the data and the critical common-sense interpretation to allow new insights to emerge (Kondracki, Wellman, & Amundson, 2002). This is a suggested approach when existing research literature on a phenomenon is limited (Hsieh & Shannon, 2005). We asked analytic questions such as “What do the interview statements express about restraint?” and “What do the interview statements express about the healthcare providers’ own perspectives on restraint?” These questions enabled us to develop latent and manifest interpretations of the participants’ perspectives and to merge the subcategories into overarching themes. All co-authors engaged in discussions on the final interpretations. Such interpretations go beyond a structuring of the manifest meanings of what is said to a deeper meaning and a more critical interpretation of the text (Kvale & Brinkmann, 2009).

## Results

The analysis resulted in three main themes: (1) disparate views on the concept of restraint and restraint use, (2) ways to limit the use of physical restraint and its negative consequences, and (3) experience with the role of parents and their influence on restraint. Perspectives from both healthcare professions were represented in all the main themes and had many similarities. When one profession differed from the other, this is noted specifically in the results.

### Disparate views on the concept of restraint and restraint use

The interviews showed that participants did not agree about many of the different core aspects of restraint, such as what to call such actions, how frequently restraint episodes were, and the consequences of restraint. Instead, healthcare providers held different perspectives and definitions of the phenomenon that “restraint” usually refers to. Furthermore, they also varied regarding how much emotion and interest they attached to the phenomenon. “Holding” was the most commonly used term to describe all kinds and degrees of physical force used during the procedure. One participant refused to use the word “restraint” because she felt that the PVC was necessary and in the child’s best interest. Further she noted that “restraint” was a very negative term that should not be used in these situations. Several nurses and physicians used the term “restraint” and explained how previous PVCs had escalated into restraint.

Nurses’ and physicians’ lack of shared understanding of restraint could be related to the fact that restraint was not commonly discussed among nurses and physicians on the unit, within as well as across the professional groups. For example, nurses and physicians lacked a shared understanding of how often they said that restraint was used. Some said restraint hardly ever happened, while other participants said it was almost an everyday occurrence. Many nurses and physicians said they felt terrible when a child was held and expressed pain, anxiety, and fear and appeared to not understand the need for the procedure. Two inexperienced nurses were quite affected when talking about how difficult and demanding it could be to use restraint. One of the most inexperienced nurses said that she sometimes felt she was participating in an assault.

The extent to which the participants allowed the issue of restraint to influence their clinical decision making also varied. For example, most physicians expressed that to be able to make a rational decision about the need for PVC, one could not let a consideration about restraint enter one’s judgment. One physician said: “But you cannot take it [restraint] into consideration either because then that will affect whether you think the child should have a venous access, which is purely a medical decision.”

The nurses and the physicians had little thought about whether the use of restraint was legal according to regulations. Some doubted whether it was legal, but lacked precise accounts. Some participants who had participated in the recorded PVCs with a lot of use of force took a defensive position when they commented on their actions. Phrases such as “I was just thrown into it,” “I am really quite inexperienced,” and “Usually, I prepare them much better, and just not in the hallway” were used. One physician also said that the particular situation that was video-recorded was a one-time-only situation.

Most healthcare providers shared the opinion that the parents’ role was to hold the child on their lap during the procedure, “to be there for the child,” to comfort, and to hold their arms around him or her. Some stated that this could be difficult for some of the parents but had different opinions about whether this meant that parents participated or ought to participate in restraint or not. Some negotiated that “hard holding” was not the task of the parent’s but of the healthcare providers, and consequently parents did not participate in restraint. Others said that parents should participate in the restraint to signal the importance of the procedure to the child, and that parental participation was not a subject for discussion. Healthcare providers disagreed about using the label of restraint on the parents holding of their child.

### Ways to limit the use of physical restraint and its negative consequences

Restraint was mostly seen as something that was necessary and inevitable because preschool children had a natural disposition to resist medical procedures and strongly disliked being held still. There were doubts but nurses felt they had few alternatives. Some of the physicians described PVC as a small technical task, which was not a big deal and usually quickly forgotten by the children. However, most of the participants explicitly or implicitly expressed that restraint with its negative consequences was something that should be limited as much as possible, and they were concerned about possible causes of restraint. Although the participants described restraint as a necessary evil, there was consensus that measures should be taken to reduce or eradicate the influence of possible causes of coercion.

The nurses and physicians asserted that they never used more force than necessary and that they constantly adjusted the forcefulness of their holding to the child’s resistance. The participants said that they held the child’s limbs only to prevent the child from withdrawing the leg or the arm. If there was a risk that the child’s resistance could ruin a PVC attempt due to movements that interfered with fixation of the PVC, they considered it better to restrain quite forcefully, to reduce the number of attempts.

A common approach was to limit the number of attempts each healthcare provider could make to perform successful PVC. With little variation, a healthcare provider stopped after three unsuccessful attempts at PVC and let a colleague take over. The reasons they gave for this practice was that after some failed attempts they lost faith in their own abilities to perform the procedure, but more importantly, they wanted to show the parents that they were responsible healthcare providers who did not “use a needle just for fun.”

The nurses also emphasized spending time to get connected with the child and to prepare the child for the sensory experience and the sequence of the different steps of the procedure. Preparation was considered a very sensitive matter, because they balanced between not worrying the children about the sensation of pain while simultaneously not underestimating it: “I do not try to deceive them. That is lying, and they will feel disappointed if the situation turns out bad.” In addition, they considered it important to successfully cannulate the vein during an early stage of the procedure to maintain an initial trustful connection with the child and the parents.

### Experience with the role of parents and their influence on restraint

During analysis, the perceived role of the parents of the resistant child emerged as a key to minimizing and preventing the use of restraint. In the experience of most of the participants, the parents’ emotional reaction to their child’s resistance was challenging. One physician said, “Well, often you think that the situation is problematic not because of the child but because of the parents.” The healthcare providers claimed that restraint could be avoided if they managed to keep the parent(s) calm and cooperative enough to endure the situation. This was referred to as parent(s) and healthcare providers being “on the same side.” One nurse nuanced this by saying that this did not mean that the child was on “the other side” in terms of an opponent, while most seemed to mean that it was impossible to make the child cooperate if the parents did not.

Healthcare providers felt that the parent’s strong emotions, such as tears, anger, insecurity, or doubts during a procedure, affected the child in such a way that the child’s tears, anger, and resistance increased. This tended to escalate, leading to more emotions, insecurity, and doubt in the parent, which made it more difficult for the child to cope. This escalation of family emotions also made the conditions and context for performing the procedure chaotic and difficult. Healthcare providers acknowledged that the situation could be difficult for parents but could feel caught in these escalating situations. The participants concluded, however, that there was little they could do when the situation “got out of hand.”

One physician said, “They [the parents] want to participate but are still reluctant,” indicating that some parents did not actively participate during the procedure. Several nurses and physicians said these parents seemed carried away by the child’s crying and emotions. After viewing the recorded PVC, one physician said, “[The mother] was very concerned about the child’s views. I feel maybe that she should have been a bit more decisive and told the child that this is something we have to do.” A nurse further connected the lack of decisiveness to the parenting: “I feel that all the choices and all the possibilities they have to negotiate and discuss themselves in and out of things make children very unsure and unsafe.” Most healthcare providers thought that stricter parenting communicated confidence and safety to the child because, as one said:

When the mother has no restrictions, then the child does not know what is right or wrong. The child makes its own decisions. If there had been restrictions at home and then the mother had said that this is something we need to do, and it is going to be like this and this and then we’re done, then I think it would be much better for the child.

Healthcare providers felt that calm and confident parents prevented an escalating situation that required much restraint.

Participants expressed that it was important to help parents remain rational and cooperative so that healthcare providers, in a controlled way, could provide the child with intravenous access. One participant said, “There are problems when the parents get too emotional. It is about informing the parents well enough.” The strategy they deemed important was: (1) to explain why the PVC was necessary and (2) to give information about the technical steps in the procedure and accompanying sensations. This information was experienced as sufficient in most situations. The participants reasoned that if parents understood how important the procedure was for the child’s medical treatment needs, they would retain this understanding during the procedure, even if restraint was needed. One nurse said that if she sensed that the parents were reluctant, she sent a person to the parents with more power to underscore the importance of the procedure and convince the parents—typically a physician. However, when viewing the recorded situation on tape where this was done, one physician concluded that the information given to convince the parents did not seem to make a difference: “It is just like the parents do not make connections between what you explain that you are going to do and what you actually do.”

Most healthcare providers felt it was very difficult to influence unconfident parents to behave more consistently toward their own children. The healthcare providers tried to act confidently and influence the parents by being calm and by talking in a decisive tone. One nurse put it like this: “The less confident the parents present themselves; the more confident I try to present myself.” A physician stated:

So, it is therefore nice to do the assessment [of the child with the parents present] before the PVC. If you notice that they let the children rule and choose, then you might before the PVC say that when we are doing this; it is important that this is something that you cannot let your child choose. We must both signal that when we are doing this we are both decisive.

## Discussion

In this study, we analysed interviews with nurses and physicians. Using perspectives from symbolic interactionism, we identified how the healthcare providers attached meaning to things and people in the interactions—for example, the way they defined the concept of restraint, their considerations on restraint, and their views on the role of the parents of the resisting child. We identified that participants used certain symbols or terms to describe their practice. For example, most healthcare providers preferred the term “holding,” and some resisted other terms such as “coercion” and “restraint.” “Holding” represented a shared meaning among healthcare providers. This has previously been identified among nurses but not among physicians (Brenner et al., 2014). For the healthcare providers, “holding” and a label such as “immobilization” may have fewer problematic professional and moral connotations than “restraint.”

A naming-discussion about using restraint on children is found in research literature, guidelines, and opinion papers (Bray et al., 2016; Brenner et al., 2014; Royal College of Nursing, 2003, 2010). Naming or symbols in use are not irrelevant. Within SI, the ability to name something signifies that one can name, and thus that one is in the position to signal to oneself and to others how the actions should be understood. For example, the term “holding” signals that this is a “neutral” or “caring practice” that in turn may contribute to the understanding of restraint as a natural or uncontroversial part of medical procedures performed on preschool children. Healthcare providers may be aware of the expectations to act in a caring way that others hold them to and may therefore choose specific labels. Conversely, the term “restraint” signals that this is a “coercive practice.” Our results support Page and McDonnell’s (2013) description of the restraint as an “uncontested practice.” When the term restraint is used for actions with children, it can hopefully result in a more governed and regulated practice than when a child has “just been held.”

If the children and parents could disagree with the healthcare providers’ use of “holding,” they would perhaps name the actions differently. The preschool children’s views and opinions of the procedures can be difficult to obtain, but their expressions of resistance can indicate that the situations with restraint are not neutral to them (Svendsen et al., 2015). The naming of actions is relevant because it may signal the level of force needed to accomplish a procedure (Darby & Cardwell, 2011; Graham & Hardy, 2004; Hart et al., 2008) and may also reflect the healthcare providers’ moral evaluation of coercive practices as unproblematic, as a necessary evil, as something we should prevent and mitigate to a further extent, or as deeply problematic. Regardless of the amount of force used, the child and parents may experience the situation as more intrusive and distressing than the term “holding” indicates.

In this study there was a tendency to avoid speaking about the controversial aspects of the coercive practices that the healthcare providers participated in and to evade responsibility. Many expressed that restraint was something that “just happened” in the situation and was, to a lesser extent, something they thought they could plan for. When restraint is considered something that “just happens,” the protection of common healthcare values such as voluntariness, showing conscientiousness, and discernment in care (Beauchamp & Childress, 2013) may not be equally important because it is beyond healthcare providers’ control. Restraint is often described as a necessity and rarely critically discussed. The different opinions on restraint and the strategies used to minimize it indicate that most professionals view restraint as problematic and think that there are many ways to prevent the use of restraint and mitigate possible negative consequences.

Some healthcare providers doubted whether the use of restraint was legal. Restraint in paediatric care is less explicitly regulated compared to restraint in adult healthcare (Sacks & Walton, 2014). Hence, healthcare providers may doubt when or whether restraint is acceptable or can be openly discussed as part of clinical practice. The lack of explicit regulations may also imply that restraint in paediatric healthcare is viewed, valued, and approached differently than in adult healthcare. This can be problematic on behalf of the children undergoing everyday medical procedures that involve various degrees of restraint because the search for and the use of alternatives to restraint may be hampered or overlooked. Page and McDonnell (2013) argued that healthcare providers need to revive a common definition of “good” around the actions of holding, which can hopefully lead to holding skills being more clearly defined and evidence-based. The restraint addressed in this study is neither recognized nor regulated, and is thus “informal.” The informal use of restraint may create a no-man’s-land where children are likely to be forcefully held with little guidance to underpin actions.

The results showed that discussions about restraint were almost non-existent. Some participants denied the existence of restraint, viewed it as an inevitable or necessary evil, or thought that it should not be deliberated when PVC was considered. This may have the unwanted side-effect of silencing a professional discussion and exploration of restraint (Kangasniemi et al., 2014). Furthermore, defining restraint as something very negative (and thus almost non-existent) may also have unintended side-effects. Bray, Snodin, and Carter (2015) suggested that over time the emotional upset of children during medical procedures can become an accepted and expected part of practice and be regarded as something that is not necessary to mitigate or prevent. It means that procedures can be completed despite a child’s upset and lack of cooperation (Bray et al., 2016).

Pearch (2005) and others has called for discussions on restraint during medical procedures (Bray et al., 2016, 2015). When there is a lack of professional consensus, personal meanings and reasoning are ascribed to the situation and may result in differing priorities and actions (Blumer, 1969). Different priorities and actions can be problematic if they are arbitrary and reflect a lack of professional attention and openness about restraint, that is, at least partially a random and unreflective practice during procedures and often neither described nor justified. Lack of guidance and scientific attention combined with conflicting interests and values may result in insecurity, individual dogmatism, a lack of shared discussion, and a lack of shared language and terminology.

An important finding in this study was the meaning that healthcare providers assigned to parenting style and parental responsibility for the use of restraint. This adds to discussions identified in earlier studies on restraint (Brenner et al., 2014; Kangasniemi et al., 2014; Kirwan & Coyne, 2016), where studies on distress and pain have found that what parents say and do clearly affect children’s ability to cope with the procedure (Salmon, 2006; Salmon & Pereira, 2002). McCarthy et al. (2010) investigated factors affecting children’s responses to PVC and concluded that parental expectations of distress and distractive communication influenced the children’s level of distress. As pointed out by the healthcare providers in our study, parents’ reluctance to actively take part in the medical procedure can help explain the use of restraint. It is therefore vital to further explore explanations for parental reluctance and lack of consistency in these situations. Alternative explanations proposed in the literature are that reluctance seems to be a usual reaction when parents experience repeated and failed PVC attempts on their child (Svendsen et al., 2016), or that parents involved in restraining their children can feel that they are letting their child down (Alexander et al., 2010). Parents’ participation is taken for granted (Hallström et al., 2002), and our results imply that the triple role of comforter, consenter, and applier of restraint seems to be very challenging for parents of newly admitted children.

Since actions according to SI are based on an assigned meaning, the participants’ meaning assigned to emotional parents and their ability to be consistent can help to explain why healthcare providers’ main strategy was to stress the importance of the procedure and focus on the explanation of steps in the procedure. Preparation and information about the procedure are important to help the child cope and cooperate (Jaaniste, Hayes, & von Baeyer, 2007; Kolk, van Hoof, & Fiedeldij Dop, 2000). However, it can be difficult for parents to prepare themselves for eventualities such as multiple restraint episodes during a procedure if this is not explicitly addressed (Svendsen et al., 2016). Lack of communication and negotiation between healthcare providers and parents can result in a lack of parental involvement when restraint is used unexpectedly (Corlett & Twycross, 2006). Our results support the notion that healthcare providers need to communicate more openly with parents (Hallström & Runeson, 2001; Hallström et al., 2002), and we suggest that education related to restraint should be included in the preparation of parents.

### Limitations

This exploration comes with some specific limitations that need further consideration. Some of the nurses and physicians on the unit declined to participate in the study. One reason could be that the word “restraint” was used in the oral orientation before the study started and in the written consent form given to the participants. This may have caused an unintended lack of interest in the study because restraint was possibly an unfamiliar and negative normative concept for some participants. The use of this concept could have made it less desirable for some to participate in the study. We considered that we had recruited enough participants to obtain information-rich accounts from those who consented to participate. Talking about potentially ethically challenging experiences and possibly illegal practices can be difficult, especially when a video-recording is involved. This may have formed the participants’ ability to tell their own stories. The interviewer was a paediatric nurse, representing one of the professions interviewed, and unknown professional power relationships may have affected the interviews in ways difficult to fully comprehend. This could have influenced the two groups of professionals differently. Although the researcher emphasized reflexivity when preparing for interviews, she could have unintentionally influenced the participants’ deliberations regarding the use of restraint. We chose to analyse the interviews of the physicians and nurses together leading to a focus on their common views. However, we acknowledge that there could be difference between the professions that could help better explain their participation in restraint practice. This should be further explored in future research. The malfunction of the technology is a limitation and could have improved the usefulness of the video recall procedure.

## Conclusion

This study explored restraint related to performance of a common medical procedure—insertion of PVC performed on newly admitted pre-schoolers in somatic hospital care. There was great variation in the participants’ understanding of the concept of restraint. This variation was mirrored in a lack of systematic handling of restraint on the unit, apart from the routine of stopping after three missed attempts before a co-worker took over. Although healthcare providers disagreed on the parents’ role during medical procedures, they considered the actions of parents to be very important regarding whether a situation escalated into restraint or not.

Restraint during medical procedures is used in clinical practice in children’s hospitals. However, it is problematic for children, parents, healthcare providers, and the services if challenges related to restraint are neglected. We suggest that healthcare providers should initiate a brief debriefing after each incident to examine process, outcome, and experiences. Such sessions could help with refining and making processes better. More research is needed on how to better communicate with colleagues, children, and their parents concerning restraint and how to avoid restraint. Furthermore, future research should explore the actions used throughout the continuum between voluntariness and forceful physical actions in actual use.

It is important to be able to develop and evaluate targeted interventions to develop alternatives that reduce the use of restraint with children of all ages. Instead of restraint being something that “just happens” or escalates in certain situations, there is a need for awareness, openness, and debate to explore further alternatives to develop efficient strategies to minimize the use of restraint. This means that nurses and physicians working in paediatrics need orientation to the use of restraints and holding procedures and ways to discuss process and importance with parents and children. Evasion of responsibility and lack of discussion may contribute to hindering a reduction of the use of restraint in paediatric units. The results of this study may facilitate more informed and reflective discussions of restraint and contribute to higher awareness of how restraint comes about in clinical practice and thus impact the clinical care of children.
